# Deep neural network for discovering metabolism-related biomarkers for lung adenocarcinoma

**DOI:** 10.3389/fendo.2023.1270772

**Published:** 2023-10-25

**Authors:** Lei Fu, Manshi Li, Junjie Lv, Chengcheng Yang, Zihan Zhang, Shimei Qin, Wan Li, Xinyan Wang, Lina Chen

**Affiliations:** ^1^ College of Bioinformatics Science and Technology, Harbin Medical University, Harbin, China; ^2^ Department of Radiation Oncology, The Fourth Affiliated Hospital of China Medical University, Shenyang, China; ^3^ Department of Respiratory, Second Affiliated Hospital of Harbin Medical University, Harbin, China

**Keywords:** lung adenocarcinoma, deep neural network, metabolite-mRNA interactions network, biomarkers, risk model

## Abstract

**Introduction:**

Lung cancer is a major cause of illness and death worldwide. Lung adenocarcinoma (LUAD) is its most common subtype. Metabolite-mRNA interactions play a crucial role in cancer metabolism. Thus, metabolism-related mRNAs are potential targets for cancer therapy.

**Methods:**

This study constructed a network of metabolite-mRNA interactions (MMIs) using four databases. We retrieved mRNAs from the Tumor Genome Atlas (TCGA)-LUAD cohort showing significant expressional changes between tumor and non-tumor tissues and identified metabolism-related differential expression (DE) mRNAs among the MMIs. Candidate mRNAs showing significant contributions to the deep neural network (DNN) model were mined. Using MMIs and the results of function analysis, we created a subnetwork comprising candidate mRNAs and metabolites.

**Results:**

Finally, 10 biomarkers were obtained after survival analysis and validation. Their good prognostic value in LUAD was validated in independent datasets. Their effectiveness was confirmed in the TCGA and an independent Clinical Proteomic Tumor Analysis Consortium (CPTAC) dataset by comparison with traditional machine-learning models.

**Conclusion:**

To summarize, 10 metabolism-related biomarkers were identified, and their prognostic value was confirmed successfully through the MMI network and the DNN model. Our strategy bears implications to pave the way for investigating metabolic biomarkers in other cancers.

## 1 Introduction

Lung cancer is a significant public health concern as evidenced by its high morbidity and mortality rates (1). Among its various subtypes, lung adenocarcinoma (LUAD) is the most prevalent, accounting for approximately 40% of all cases (2). Metabolic alterations in LUAD are crucial for its diagnosis, prognosis, and treatment response (3). Despite advancements in our understanding of LUAD’s pathogenesis and development of therapeutic strategies, it remains an aggressive and deadly tumor type. Therefore, the identification and development of prognostic metabolism-related biomarkers for predicting outcomes in LUAD bear clinical significance (4).

Biomarkers have emerged as valuable indicators for the timely diagnosis, prognosis, and prediction of treatment responses in LUAD. These biomarkers reflect a diverse range of molecular alterations, including genetic expression patterns (5). Several studies have attempted to investigate the relationship between biomarker expression and LUAD. For instance, elevated expression levels of PD-L1 have been associated with worse prognosis and reduced survival in lung adenocarcinoma patients (6). PD-L1 expression may serve as a potential predictive biomarker for response to immunotherapy and can help guide treatment decisions. High expression of certain receptor tyrosine kinases, such as the epidermal growth factor receptor (EGFR) has been identified in subsets of patients with LUAD and has been proven effective as targets for specific TKIs (7, 8). Altered expression of microRNAs (miRNAs) has been implicated in the development and progression of LUAD (9, 10). Assessment of expressions of these biomarker levels is important in selecting the most appropriate targeted therapy approach (11, 12).

Cancer, a metabolic disease, arises from alterations in metabolism triggered by genetic or non-genetic signals (13). Tumor cells exhibit distinct metabolic characteristics, including increased proliferation and resistance to apoptosis. As tumors actively manipulate metabolic systems to sustain their growth, targeting their metabolism is a promising approach for personalized cancer therapy (14, 15). Tumor cells often switch their metabolism from mitochondrial oxidative phosphorylation to glycolysis, a phenomenon known as the “Warburg effect.” This provides energy and building blocks for tumor cell division, growth, and adaptation to oxidative stress (16). As tumor cells need to adapt their metabolic pathways to support their rapid growth and energy demands, they undergo metabolic reprogramming, a hallmark of cancer (17). Metabolic abnormalities contribute to the development and progression of cancer through the interactions between specific mRNAs and metabolites, referred to as metabolite-mRNA interactions. Metabolic pathways are crucial for tumor progression and survival; therefore, they have garnered significant research attention in the study of LUAD (18). Cao MDT, L.J., Boulanger J, et al., found that altered metabolic processes, such as increased glucose consumption, dysregulated lipid metabolism, and abnormal amino acid utilization occur commonly in LUAD cells. Understanding the intricacies underlying these metabolic alterations can provide valuable insights into the development of effective therapeutic strategies (19). Recently, Ksenia M. Shestakova et al., showed that the combination of metabolomics and cutting-edge bioinformatics is a practical tool for the accurate diagnosis of patients with non-small cell lung cancer (NSCLC) (20, 21). The study examined the relationship between metabolites and NSCLC and its original conceptualization offers a novel perspective on studying the connection between NSCLC and metabolites.

In the biomedical field, with the introduction of high-throughput technology, the amount of biomedical data, including genomic, metabolomic, and proteomic has massively accumulated (22). By storing, analyzing, and interpreting these impressive amounts of biomedical big data, it is possible to better understand human health and illness (23, 24). A type of deep learning and artificial intelligence, deep neural network (DNN) models have emerged as a potent tool for research in several fields of biology (25–27). Compared to classical machine learning techniques, deep learning has many advantages, such as strong self-learning capabilities and excellent generalization ability (28). Algorithms based on deep learning created from artificial neural networks are promising for identifying patterns and extracting features from large amounts of complex data to obtain biomarkers with clinical prognostic value (29).

Despite significant advances in biomarker identification, elucidation of metabolic pathways, and utilization of bioinformatics and machine learning techniques, several challenges remain. One of these is the identification of reliable biomarkers with high sensitivity and specificity (30). Integrating multi-omics data and utilizing DNN models is necessary to find reliable biomarkers for improving the accuracy of cancer diagnosis and prognosis prediction (31, 32). Hence, at the genomic level, the goal of our study was to identify metabolism-related biomarkers for LUAD by integrating data on gene expression, metabolite profiling, and protein interactions to construct a network of metabolites-mRNAs and mRNA interactions. We then introduced a DNN model to identify metabolism-related biomarkers for LUAD. Our findings could contribute to the advancement of metabolism-based research.

## 2 Materials and methods

**Figure 1:**
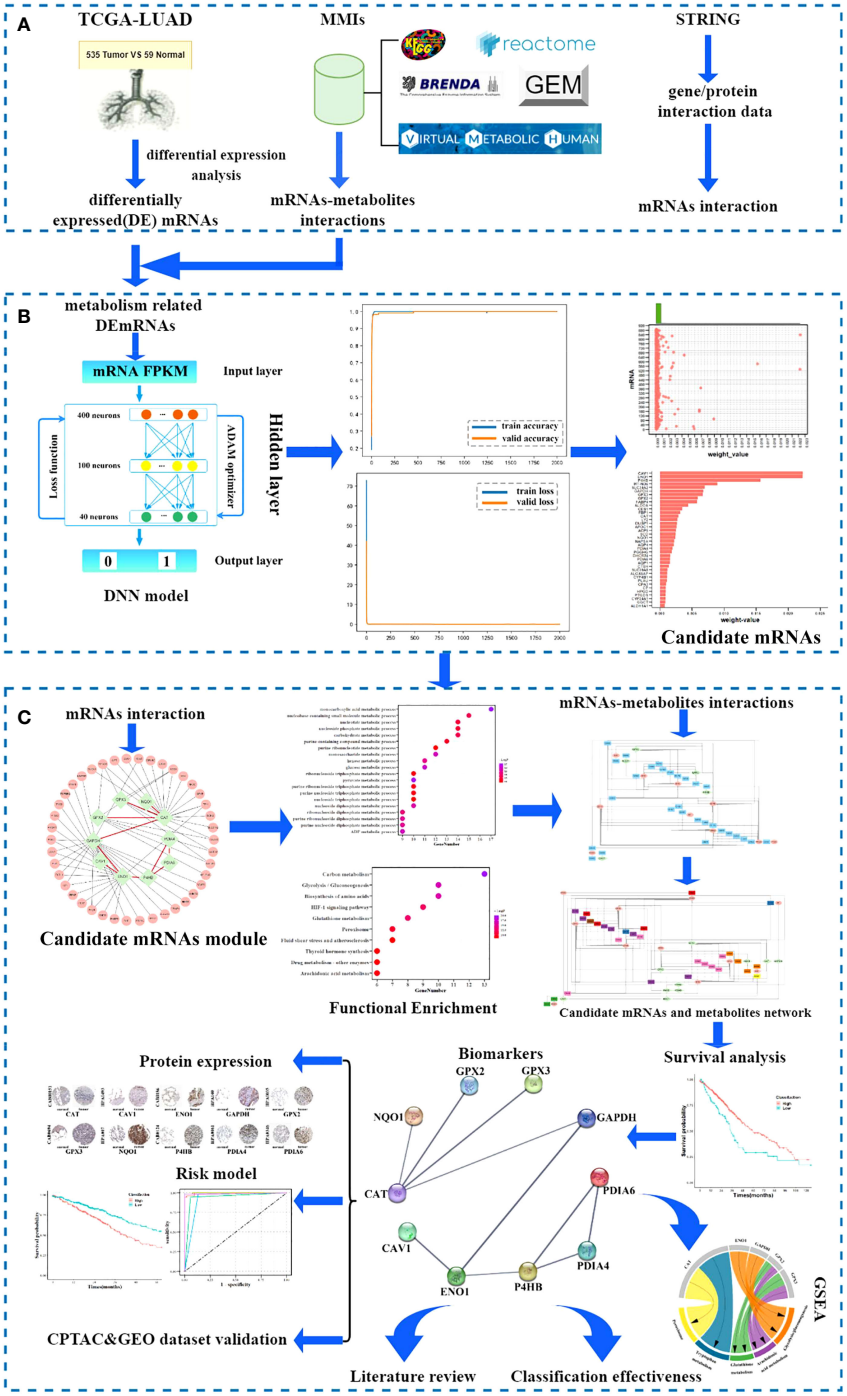
Workflow of the study. **(A)** Data sources. **(B)** Screening candidate mRNA. **(C)** Identification and validation of biomarkers.

The workflow of our investigation is shown in **Figure 1**, and the details are described in the subsequent sections.

### 2.1 Data sources

In this study, eight LUAD cohorts (**Table 1**) were obtained from The Tumor Genome Atlas (TCGA, https://portal.gdc.cancer.gov/), Gene Expression Omnibus (GEO, https://www.ncbi.nlm.nih.gov/geo/) (GSE36471, GSE42127, GSE68465, GSE72094, and GSE87340), and Clinical Proteomic Tumor Analysis Consortium data portal (CPTAC, https://cptac-data-portal.georgetown.edu/).

**Table 1:** Datasets for lung adenocarcinoma used in this study.

Data source	Platform	Follow-up information	Sample count
TCGA	Illumina HiSeq 2000	OS	594
GSE36471	GPL9053	OS	115
GSE42127	GPL6884	OS	176
GSE68465	GPL96	OS	442
GSE72094	GPL15048	OS	442
GSE87340	GPL11154	OS	54
CPTAC(mRNA)	Illumina Hiseq 4000	–	204
CPTAC(protein)	Tandem mass tags	–	214

OS, Overall survival; -, No Overall Survival.

RNA-Sequence (Seq) and clinical data from 594 samples of LUAD (containing 535 tumor tissues and 59 non-tumor tissues) were acquired from the corresponding TCGA cohort. **Table 2** lists the patients’ clinical characteristics. Symbol and gene type attributes of RNA-Seq data were annotated using the Ensemble database. According to the gene type attribute, mRNAs were extracted.

**Table 2:** Clinical characteristics of the patients with lung adenocarcinoma.

Clinical characteristics	TCGA
**Patient (n)**	**594**
Age, years	
median	65
range	33–88
Sex (%)	
female	270 (45.4%)
male	317 (53.4%)
Absent	7 (1.2%)
Stage (%)	
Stage I	317 (53.4%)
Stage II	136 (22.9%)
Stage III	97 (16.3%)
Stage IV	28 (4.7%)
Absent	16 (2.7%)

For the microarray datasets (GSE36471, GSE42127, GSE68465, GSE72094, and GSE87340) generated by the Illumina and Agilent platforms, originally processed data (series matrix files) were used (33). Probe IDs were mapped to corresponding gene IDs using the platform files.

Using four different data sources, namely the Kyoto Encyclopedia of Genes and Genomes (KEGG) (34), Reactome (35), Human-GEM (36), and BRENDA (37), we retrieved metabolite-mRNAs interactions (MMIs) (38). The Virtual Metabolic Human database’s metabolite abbreviations were utilized to standardize metabolite names to the universal nomenclature. A directed MMI network was constructed (the whole network was detailed in **Supplementary Table S1**), including 31227 unique MMIs covering 1869 metabolites and 4134 mRNAs.

### 2.2 Metabolism-related DEmRNA interaction network

Fragments per kilobase of exon per million read mapped (FPKM) values were chosen as the representative measure of mRNA expression from RNA-Seq data. Using the FPKM values, “Limma” (39) was employed to identify statistically significant and differentially expressed (DE) mRNAs between LUAD and non-tumor tissues. Specifically, a t-test was utilized for evaluating differential expression. A threshold of | log2(fold-change) | ≥ 1 and a false discovery rate (FDR) adjusted p-value < 0.05 were adopted as criteria for determining statistical significance. The collection of metabolism-related DEmRNAs was determined by combining DEmRNAs with 4134 mRNAs obtained from the MMI network. Using the tool, STRING (Search Tool for the Retrieval of Interacting Genes) (https://string-db.org/) (40) with a confidence level >= 700, a metabolism-related DEmRNA interaction network was constructed.

### 2.3 Candidate mRNAs

#### 2.3.1 Metabolism-related mRNA DNN model construction

The Google TensorFlow 2.0 architecture was used to generate a fully connected DNN model with numerous hidden layers, an output layer, and an input layer. Hence, we built a metabolism-related mRNA DNN model using the Google TensorFlow 2.0 architecture, comprising an input layer, three hidden layers, and an output layer, following a previously described workflow (41). The features of the DNN model were the FPKM values of the metabolism-related DEmRNAs. The output layer with a label of 1/0 indicated if the sample was cancerous or not. Given the small sample size, the Adaptive Moment Estimation (ADAM) optimizer with default Ker as parameters was selected. The loss function of binary cross-entropy was applied. The DNN model’s performance was influenced by three parameters related to model training, including batch size, number of epochs, and learning rate. Model training requires multiple rounds of learning. The learning rate was considered when randomly selecting a batch of training sets in each round. A larger batch size results in faster model convergence but has weaker generalization ability. Therefore, the initial values for batch size and epoch were set to 16 and >= 1000, respectively, according to the sample size and number of features. When using a large batch size, a high learning rate was required to prevent underfitting, while a low learning rate was needed for a small batch size to avoid overfitting. To achieve optimal results, a learning rate of 0.0001 was set for subsequent learning cycles, and the parameters for batch size, epoch, and learning rate were continuously adjusted based on the validation accuracy curve and results of loss curve fitting.

#### 2.3.2 Candidate mRNAs screening

In the DNN model, the larger the weight, the greater the corresponding feature’s contribution. Features that contributed significantly to the DNN model were more biologically significant. Therefore, features were screened as candidate mRNAs based on the weight of the features. The arithmetic average of absolute Shapley Additive exPlanations (SHAP) values for the impact representing the importance of the feature to all samples was denoted as the weight and it was calculated using summary_plot. SHAP (42) is an approach in game theory to explain the output of a machine learning model. The SHAP values were obtained first. Assuming that the ith sample was *x_i_*, the jth feature of the ith sample was *x_ij_*, the predicted value of the model for that sample was *f*(*x_i_*), and the baseline of the entire model (usually the mean of the target variables for all samples) was *y_base_*, then the SHAP value obeyed the following equation:


$$f\left(x_{i}\right)=y_{base}+f\left(x_{i1}\right)+f\left(x_{i2}\right)+f\left(x_{i3}\right)+\cdots +f\left(x_{ik}\right)$$

Where *f*(*x_ij_*) was the SHAP value of *x_ij_*. Intuitively, *f*(*x_i_*_1_) was the contribution value of the 1st feature in the ith sample to the final prediction value *f*(*x_i_*). When *f*(*x_i_*_1_) > 0, the feature improved the prediction value and had a positive effect; conversely, it meant that the feature lowered the prediction value and had a negative effect. The impact of a feature on the machine learning model was thus represented by the SHAP value. To determine an approximation of the SHAP values for the DNN models in this study, DeepExplainer from the Python SHAP module was employed. The SHAP value of each feature on each sample was obtained using force_plot. Finally, the weight value was calculated by summary_plot based on the arithmetic average of absolute SHAP values. Candidate mRNAs were screened by generating a scatter plot for a single variable with different histograms at the upper border of the plot using the weight values of the feature mRNAs in the DNN model.

### 2.4 Biomarkers

#### 2.4.1 Biomarker identification

From the metabolism-related DEmRNA interaction network, the module of interacting candidate mRNAs and their one-step neighbors were collected. Gene ontology (GO) functional analysis (43) was conducted to identify the unique biological properties, including biological processes (BP), cellular components (CC), and molecular functions (MF). All mRNAs in the module were extracted for GO and KEGG pathway enrichment analyses, and analyzed on the metascape platform (https://metascape.org/) (44). Categories with the minimum overlap number of 3 and the hypergeometric test Benjamini-Hochberg adjusted p-value < 0.05 were selected.

Metabolism-related pathways and functional classes were chosen based on the results of enrichment analysis and the enriched mRNAs (including candidate mRNAs and one-step neighbors) were added to the MMI network to create a module of enriched mRNAs and metabolites, which was combined with the module of interacting candidate mRNAs to create a subnetwork comprising candidate mRNAs and metabolites.

Kaplan-Meier survival analyses (45) for candidate mRNAs in the subnetwork were conducted using the “survival” package in R to confirm the prognostic effect. Overall survival (OS) was defined as the time from the date of initial surgical resection to the date of death or last contact (censored), truncated at 120 months. Survival curves were drawn using Kaplan-Meier analysis and were compared using the log-rank test for assessing statistical significance. Based on the results of the survival analysis, candidate mRNAs were identified as biomarkers.

#### 2.4.2 Biomarkers’ classification effectiveness assessment

To assess the effectiveness of identified biomarkers for LUAD, 594 samples from the cohort of TCGA-LUAD were used. Traditional machine learning methods, including K-nearest neighbor (KNN) (46), Support Vector Machine (SVM) (47), Decision Tree (48), Naive Bayes (49), and Logistic regression (50) were applied for sample classification using identified biomarkers. Their performance was visualized as the area under (AUC) the receiver operating characteristic (ROC) curves.

#### 2.4.3 Protein levels of biomarkers

Images depicting protein expression in normal tissue and pathology of tumor tissue sections were downloaded from the Human Protein Atlas (HPA, https://www.proteinatlas.org/) database to determine differential expression at the protein level.

### 2.5 Validation of biomarkers

Furthermore, Kaplan-Meier survival analysis was conducted in five independent GEO- LUAD datasets (GSE36465, GSE42127, GSE68465, GSE72094, and GSE87340) to further validate the prognostic value of biomarkers. A total of 204 samples of LUAD from CPTAC comprised an independent dataset and were used to validate the effectiveness of the identified biomarkers.

A literature review was conducted by searching the PubMed database for all articles published in the English Language on the relevant topics of identification of biomarkers for LUAD and the relationship between biomarkers and metabolites.

## 3 Results

### 3.1 Candidate mRNAs

**Figure 2:**
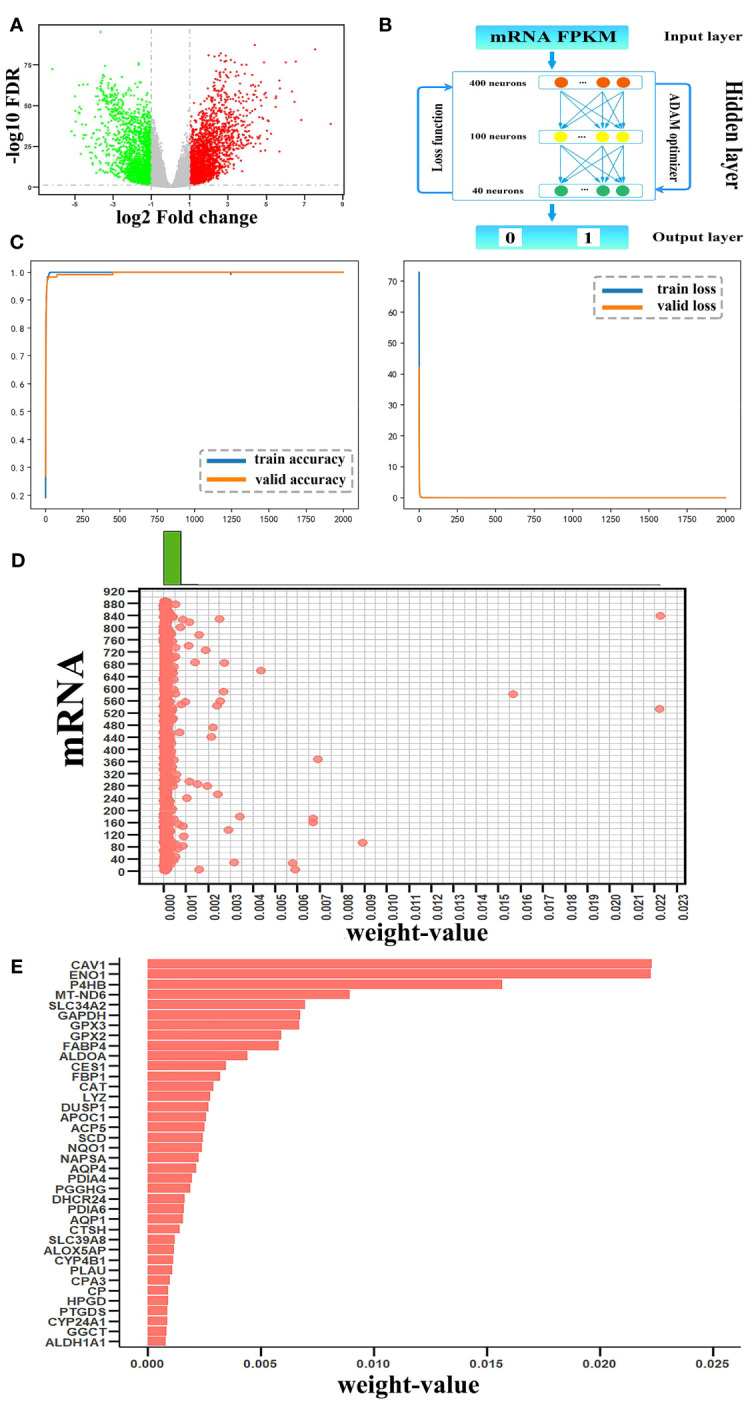
Candidate mRNAs. **(A)** Volcano plot of differentially expressed (DE)mRNAs between tumor and non-tumor samples. Red and green represented upregulated and downregulated DEmRNAs, respectively. **(B)** The structure of the DNN model. **(C)** The accuracy curve and the loss curve of the mRNA DNN model. **(D)** The joint distribution of weight values. The x-axis represents the weight-value of each mRNA and the y-axis represents 887 mRNAs, as 1–887 to indicate each mRNA. **(E)** The weight values of the top 38 candidate mRNAs.

First, in the TCGA dataset, using Student’s t-test with a false-discovery rate (FDR) < 0.05 and | log2(fold-change) | >= 1, 4376 DEmRNAs between the 535 LUAD samples and 59 non-tumor samples were extracted; among them, 2448 and 1928 DEmRNAs were upregulated and downregulated, respectively (**Figure 2A**). A total of 887 metabolism-related DEmRNAs (**Table S2**) were obtained from the overlap of 4376 DEmRNAs and 4134 mRNAs from the MMI network. Using the STRING database, a metabolism-related DEmRNA interaction network was constructed with 887 nodes and 1852 edges (the entire network was detailed in **Supplementary Table S3**).

For the metabolism-related DEmRNA DNN model, the initial input layer was set with the FPKM values of 887 DEmRNAs, three hidden layers of 400, 100, and 40 neurons, and the 1/0 label as the output layer (**Figure 2B**). The 594 samples were split randomly with 80% in the training set and 20% in the testing set. Through the output label and after setting batch size = 16, epoch = 2000, and learning rate = 0.00001, the validation accuracy curve and loss curve both conformed to the general law of deep learning. The accuracy reached 99.7% (**Figure 2C**). Thus, the regularization optimization was effective.

Python’s DeepExplainer SHAP module was applied to interpret the contribution of each mRNA to each sample in the DNN model; SHAP values were obtained using force_plot. It demonstrated that each feature contributes differently to the prediction of the model from the base value (*y_base_*) to the final fetch *f*(*x_i_*). Based on the definition of the weight of the mRNA in the DNN model, the arithmetic mean of the absolute SHAP values representing the influence of the feature on the importance of all samples were calculated by summary_plot and expressed as the corresponding weight values. To select candidate mRNAs with high contributions to DNN models, a scatterplot was generated for a single variable, and different histograms were plotted on the upper boundary of the scatterplot (**Figure 2D**). According to the distribution of mRNAs in the scatterplot, mRNAs arranged according to the weight values were mainly concentrated on two sides of the weight value of 0.00075; therefore, we chose these 38 mRNAs with weight value > 0.00075 as candidate mRNAs (**Figure 2E**). Statistical analysis showed that the top 38 mRNAs contributed 0.1558 to the total, while the remaining 849 mRNAs contributed 0.1109.

### 3.2 Analysis of the candidate mRNA module

**Figure 3:**
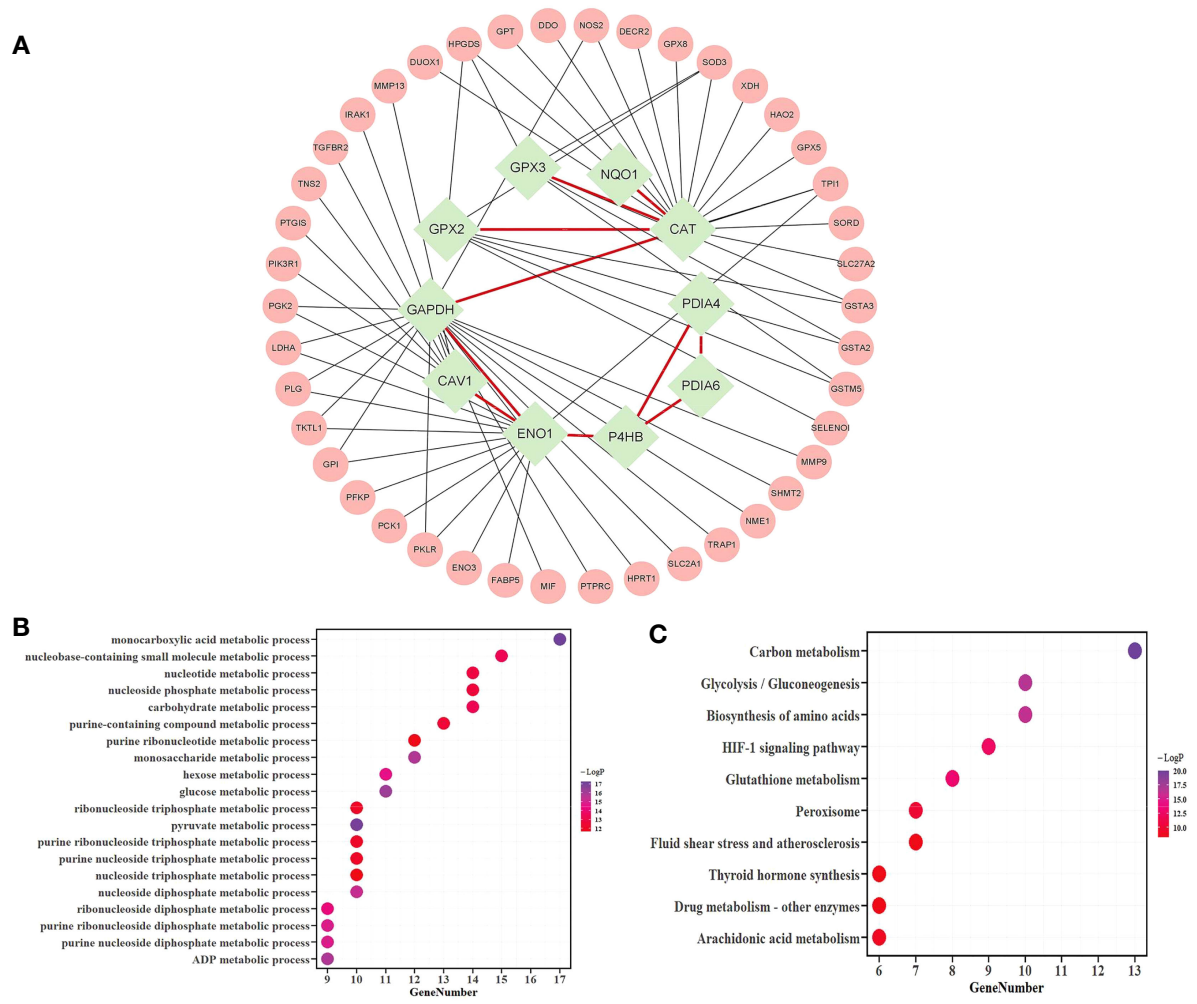
Analysis of the candidate mRNAs module. **(A)** The candidate mRNAs module. Results of the enrichment analyses of 52 mRNAs were represented in a bubble diagram; **(B)** The findings of the GO enrichment analysis for functions linked to metabolic processes, with -log_10_ P >= 10; **(C)** The results of the KEGG enrichment analysis, assuming -log_10_P >= 8.

A total of 38 candidate mRNAs were identified by differential expression analysis and DNN model screening. From the STRING database’s protein interaction data, the metabolism-related DEmRNA network of LUAD was built. From this network, 10 candidate mRNAs (CAT, CAV1, ENO1, GAPDH, GPX2, GPX3, NQO1, P4HB, PDIA4, and PDIA6) showed interactions. The metabolism-related DEmRNAs network was segmented into an interacting candidate mRNA module (**Figure 3A**) that included these 10 candidate mRNAs for interaction and the 42 one-step neighbor mRNAs that they were connected with.

The metascape platform was used to conduct functional enrichment analysis based on GO and KEGG databases for candidate mRNAs of LUAD. Categories with the minimum overlap number of 3 and the hypergeometric test Benjamini-Hochberg adjusted p-value < 0.05 were selected. Fifty-two mRNAs were identified as considerably enriched in functional classes relevant to metabolic processes by GO enrichment analysis (**Figure 3B**), and arachidonic acid and glutathione metabolic pathways were included among the top 10 of the KEGG enrichment results (**Figure 3C**).

**Figure 4:**
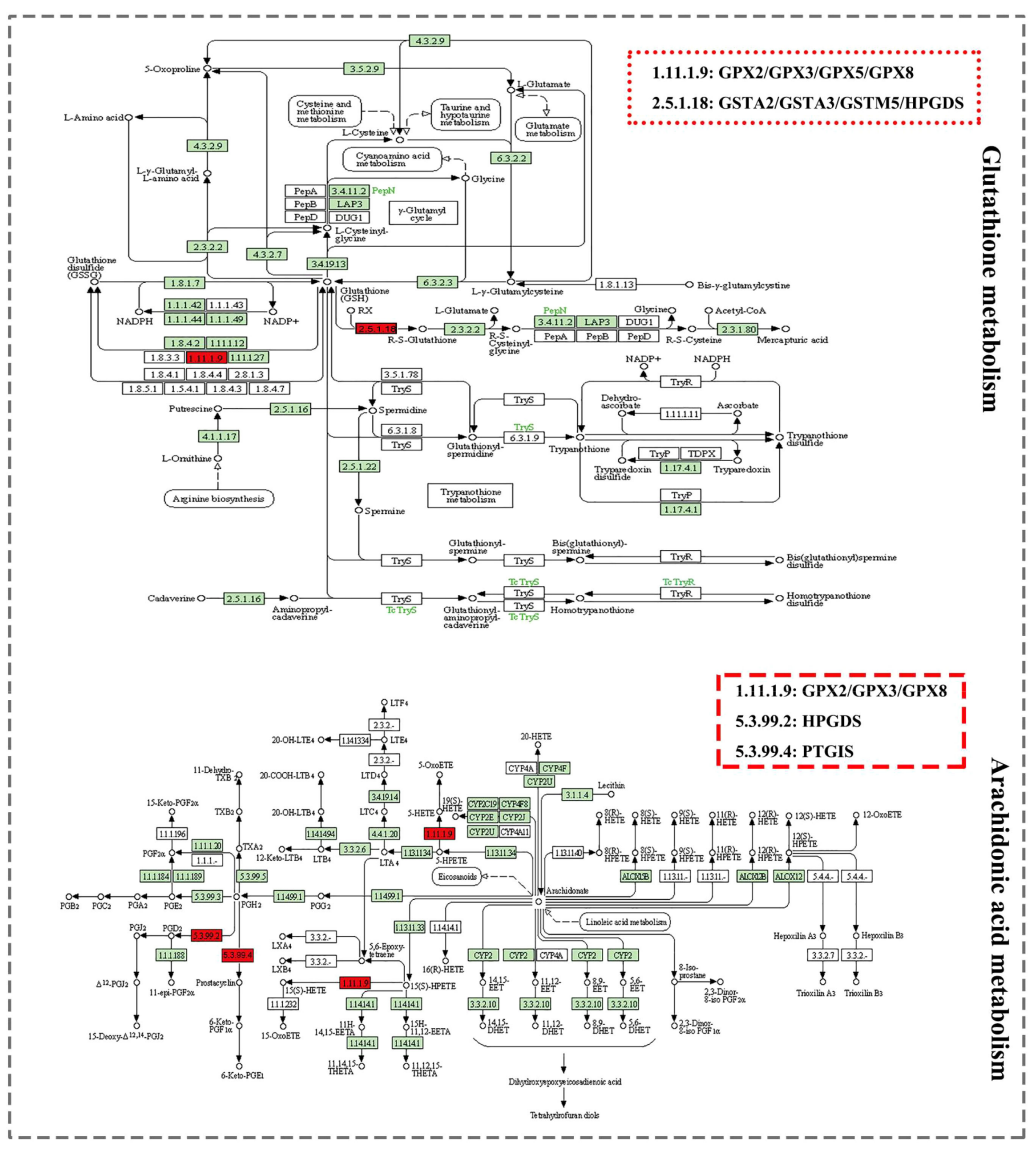
Glutathione and arachidonic acid metabolic pathways: Red tags showed enriched mRNAs.

The KEGG database showed two metabolic pathways, namely glutathione metabolism (51) and arachidonic acid metabolism (52), which were chosen for subsequent analyses (**Figure 4**). First, in the glutathione metabolism pathway, reduced glutathione (GSH) is converted to oxidized glutathione by the enzymes GPX2 and GPX3 in glutathione metabolism (GSSG). Several prevalent human diseases, including lung cancer, are partially caused by impaired glutathione metabolism. Nevertheless, GPX2 and GPX3 are engaged in the body’s metabolic mechanism for maintaining glutathione levels, which can successfully prevent lung cancer (53). In addition to being crucial for lowering inflammatory reactions, improving immunological function, and ensuring normal gene and protein expression, stabilizing glutathione metabolism also controls the proliferation and death of human cells.

In the metabolism of arachidonic acid, arachidonic acid functions through GPX2 and GPX3 to form 15(S)-HPETE (54). One of the six monohydroperoxy fatty acids generated by the non-enzymatic oxidation of arachidonic acid is 15(S)-HPETE (leukotrienes). Hydroxy fatty acid (+/-)15-HETE, which is more stable, is produced by reducing hydroperoxides. Arachidonic acid belongs to a group of bioactive substances produced by the 5-lipoxygenase pathway in oxidative metabolism, implicated in pathophysiological roles such as inflammation (55), acute hypersensitivity (56), and host defensive reactions. The lung is an important organ that is significantly affected (57). Additionally, there are three ways that arachidonic acid metabolites can influence the development and metastasis of lung cancer as follows: prostacyclin inhibits platelet-tumor cell contact; thromboxane increases platelet-tumor cell contact and thus encourages tumor cell invasion; prostaglandins' cytoprotective activity maintains the integrity of epithelial cells and affects tissues' responses to pro-tumorigenic substances, and through lipoxygenases (58).

### 3.3 Biomarkers

#### 3.3.1 Biomarkers’ identification

Twenty mRNAs, including six candidate mRNAs (CAV1, ENO1, GPX2, GPX3, NQO1, and P4HB) and 14 one-step neighbor mRNAs (GPI, GPX5, GPX8, GSTA2, GSTA3, GSTM5, HPGDS, MIF, MMP9, PIK3R1, PTGIS, PTPRC, TRAP1, and XDH) were enriched in the functional classes of glutathione and arachidonic acid metabolic pathways and metabolism-related biological processes.

**Figure 5:**
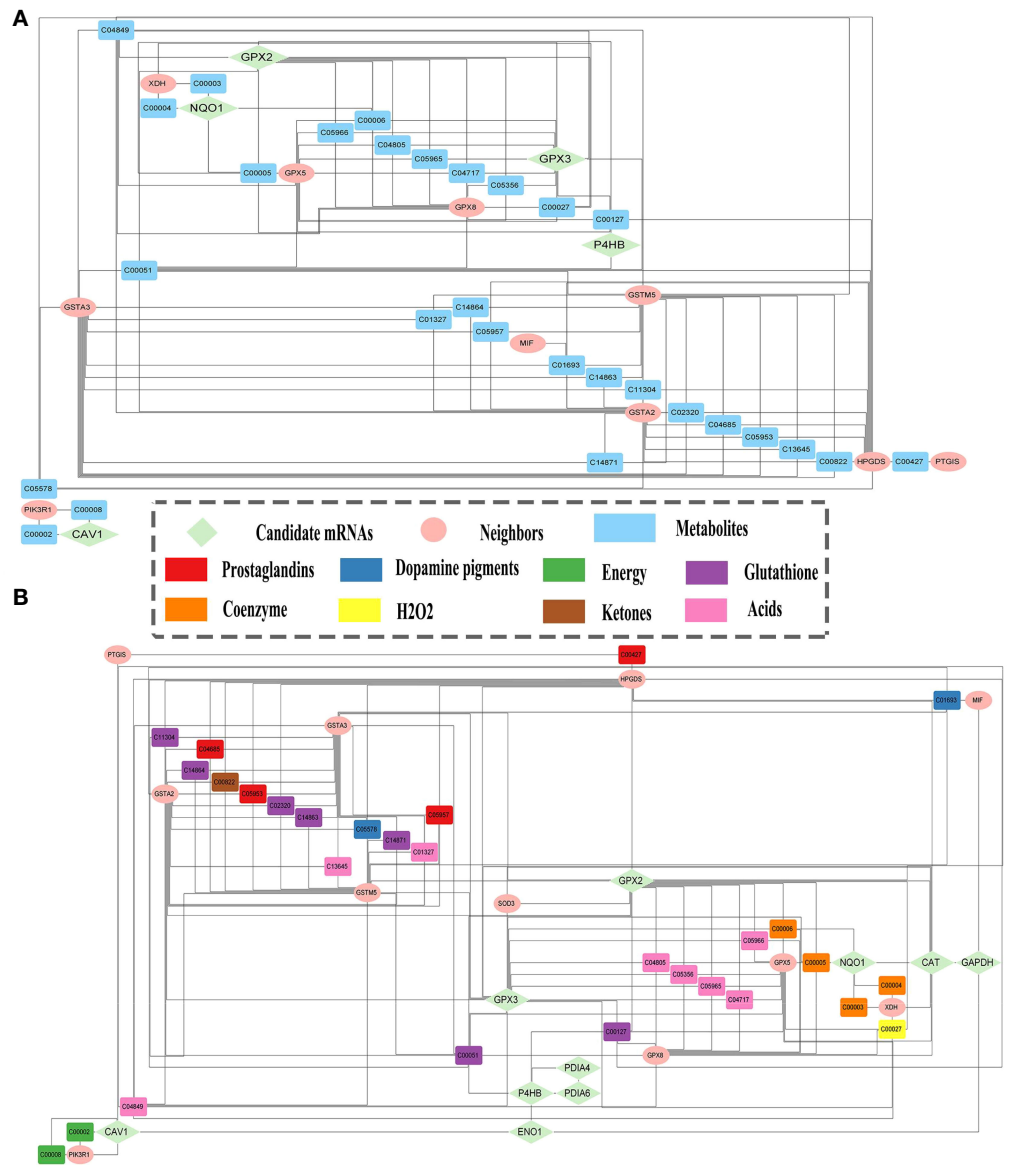
Candidate mRNAs and metabolites relationships. **(A)** The module of enriched mRNAs-metabolites. **(B)** Subnetwork of the relationships between metabolites and candidate mRNAs, and their one-step neighbor mRNAs.

A module of enriched mRNAs-metabolites was extracted from MMIs, including 20 mRNAs and 71 metabolites (**Table S4**). It was further refined to eliminate unimportant metabolites such as water, oxygen, H+, etc., and metabolites with a degree of 1, such as phosphate, xanthine, hypoxanthine, etc. Thus the refined module (**Figure 5A**) consisted of 15 mRNAs and 29 metabolites, including 5 candidate mRNAs (**Table S5**). This refined module was then combined with the module of interacting candidate mRNAs to create the subnetwork comprising candidate mRNAs and metabolites. Irrelevant mRNAs with a degree of 1 that did not contribute to the mRNA-metabolite association were removed (**Figure 5B**). Thus, the final subnetwork was constructed, including 10 candidate mRNAs together with 11 one-step neighbor mRNAs and 29 metabolites. Eight primary categories—energy, coenzymes, hydrogen peroxide, glutathione, prostaglandins, ketones, acids, and dopamine pigments—were utilized to classify the metabolites to conveniently display the types of mRNAs-linked metabolites.

**Figure 6:**
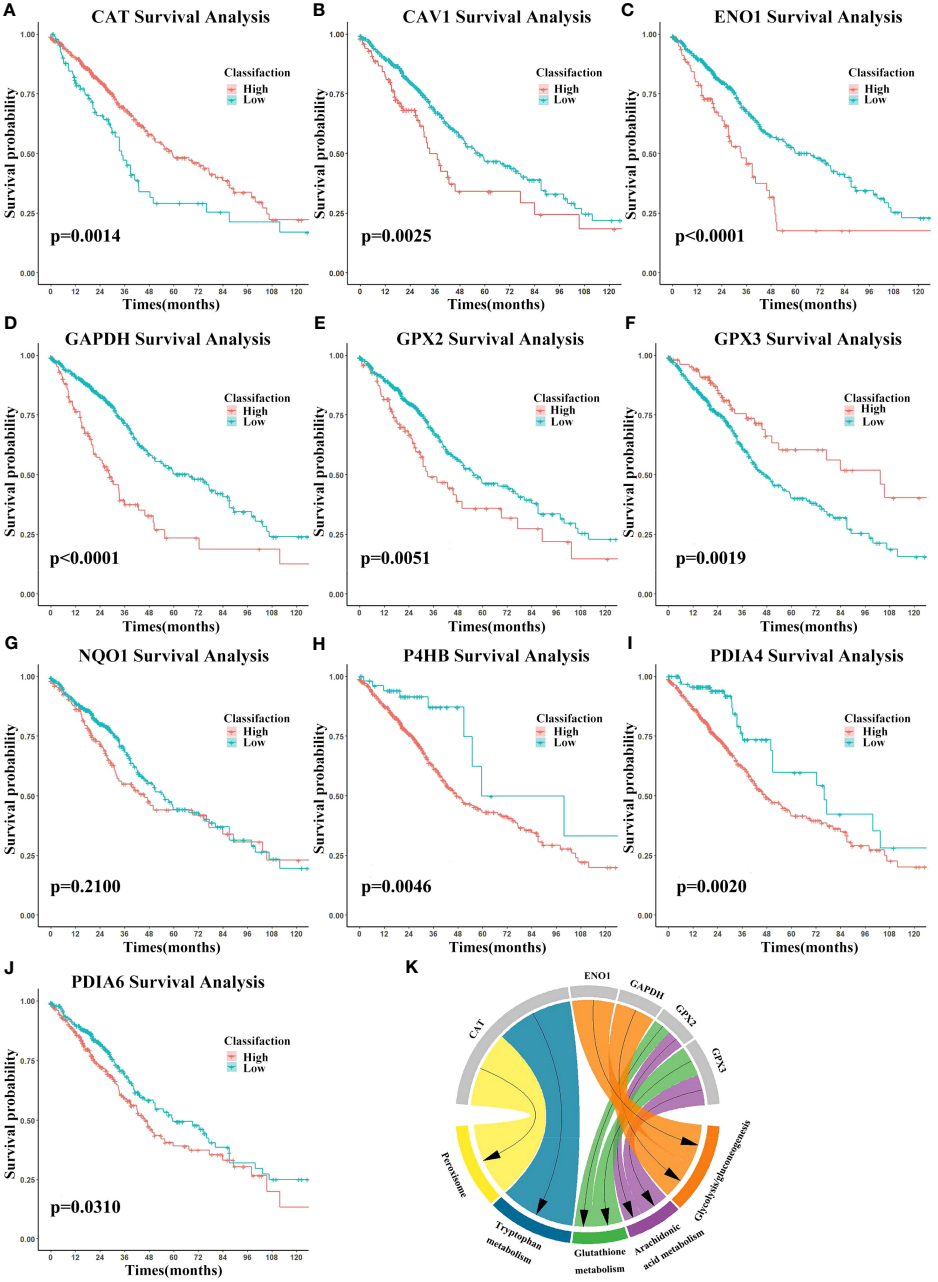
Biomarkers’ survival analysis and gene set enrichment analysis (GSEA). **(A–J)** Survival curves using biomarker expression. Survival time is on the x-axis and survival probability is on the y-axis. **(K)** GSEA results were shown on the chart.

Using the univariate Kaplan-Meier survival analysis, the predictive significance of candidate mRNAs in LUAD was assessed. The “ggsurvplot” package was used to plot survival curves, and log-rank tests were used to compare results (**Figures 6A–J**). Except for NQO1, the remaining nine candidate mRNAs had a substantial predictive ability. Next, we conducted a literature-based validation of NQO1’s prognostic outcome in LUAD (59). We found that NQO1 is a potential therapeutic target and predictive biomarker for LUAD. All the ten candidate mRNAs were identified as biomarkers.

To investigate biomarkers’ function and correlations with the cancer phenotype, GSEA (https://www.gsea-msigdb.org/gsea/) (60) was performed using the expression data. The KEGG gene set was selected and biomarkers were ranked. In cancer, biomarkers were significantly enriched for glycolysis/gluconeogenesis, while arachidonic acid metabolism, glutathione metabolism, tryptophan metabolism, and peroxisome were enriched in the normal setting (**Figure 6K**). These findings shed light on the intricate relationship between biomarker expression and metabolic processes, thereby affirming the relevance and credibility of the identified metabolism-related biomarkers.

#### 3.3.2 Biomarkers’ classification effectiveness assessment

**Figure 7:**
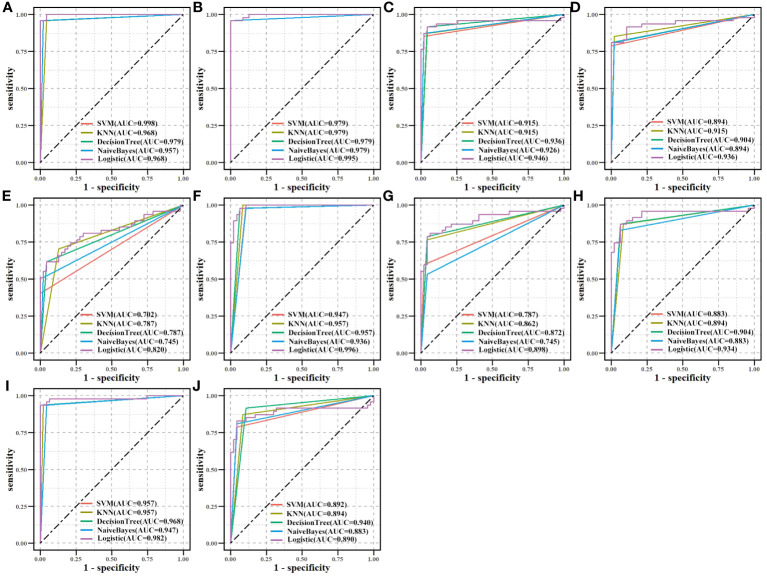
Biomarkers’ classification effectiveness assessment under using different classifier models in TCGA. **(A)** CAT, **(B)** CAV1, **(C)** ENO1, **(D)** GAPDH, **(E)** GPX2, **(F)** GPX3, **(G)** NQO1, **(H)** P4HB, **(I)** PDIA4, and **(J)** PDIA6.

We assessed the effectiveness of biomarker-based classification in LUAD using traditional machine learning techniques (SVM, KNN, Decision Trees, Naive Bayes, and logistic regression). The classification effectiveness of each biomarker is shown in **Figure 7**.* A* total of 594 samples were split into a training set and a test set in a ratio of 8:2. TPR (sensitivity) was used as the vertical coordinate and FPR (1-specificity) as the horizontal coordinate to plot the ROC curves against different critical values. Most machine learning methods had good classification performance (AUC > 0.700), demonstrating the classification effectiveness and diagnostic values of all biomarkers for LUAD samples.

#### 3.3.3 Differential expression analysis

**Figure 8:**
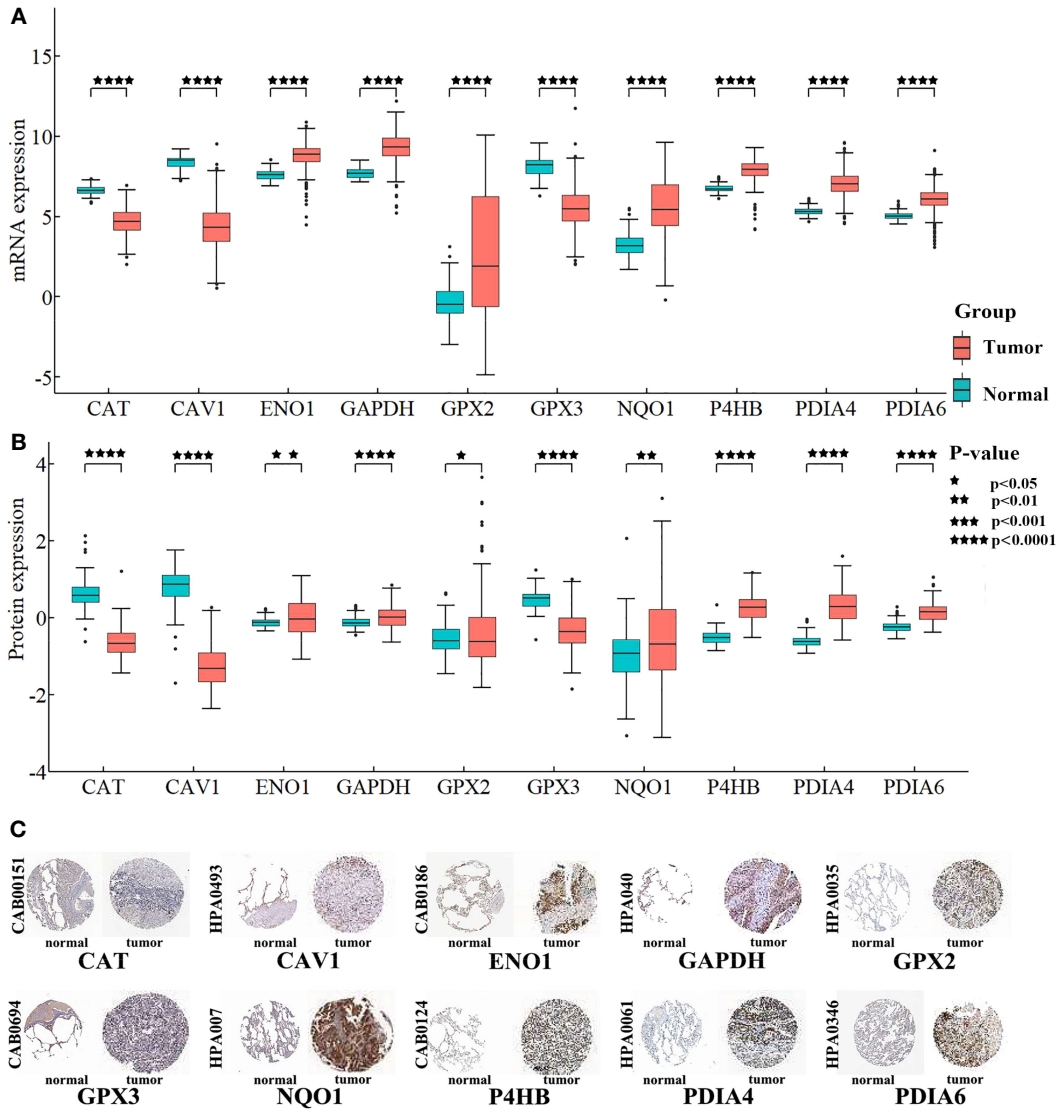
Differentially expression analysis for biomarkers. **(A)** Box plot shows the differential expression of mRNAs. The Y-axis is the biomarkers’ expression after log2 transformation. **(B)** Box plot shows the differential expression of proteins. The Y-axis is the proteins’ expression value after log2 transformation. **(C)** Proteins’ differential expression in the HPA database. The left side of the panel shows the antibody numbers.

Differential expression of the 10 biomarkers was analyzed between cancer and normal samples at the mRNA and protein level (**Figure 8**). In the CPTAC, all 10 biomarkers’ coding proteins showed differential expression, and their expressions matched their mRNA levels in TCGA. The HPA database was searched for the expression profiles of the proteins corresponding to each of the 10 biomarkers in normal tissue and tumor tissue sections. The detection of homologous antibodies demonstrated that the differential protein expression in the samples was compatible with the information in the CPTAC database.

### 3.4 Validation of biomarkers

Prognostic values for biomarkers in LUAD were validated in five independent datasets obtained from GEO (**Table 3**). The percentage of validated significant prognosis for biomarkers was more than 70% and reached up to 90% in the GSE87340 dataset. Each biomarker was validated in more than three datasets. Combining the results of the TCGA and independent GEO datasets suggested that these biomarkers were stable predictors for survival in LUAD.

**Table 3:** Survival prognosis.

	GSE68465	GSE36471	GSE42127	GSE72094	GSE87340
CAT	0.0023	0.0030	0.0157	<0.0001	0.0002
CAV1	0.0180	0.1739	0.0433	0.0022	<0.0001
ENO1	0.1045	0.0027	0.0092	0.1032	0.0023
GAPDH	0.0019	0.0023	0.0084	<0.0001	0.0001
GPX2	0.0151	0.0007	0.0031	0.0003	0.1247
GPX3	0.0423	0.0447	0.0075	0.0002	0.0053
NQO1	0.0062	0.1604	0.0793	0.0151	0.0414
P4HB	0.0902	0.0190	0.0585	0.0216	0.0122
PDIA4	0.1178	0.0368	0.0038	0.0611	<0.0001
PDIA6	0.0223	0.0400	0.0028	0.0107	0.0001
Ratio	70%	80%	80%	80%	90%

Ratio: Ratio of biomarkers with prognostic survival (p less than 0.05).

**Figure 9:**
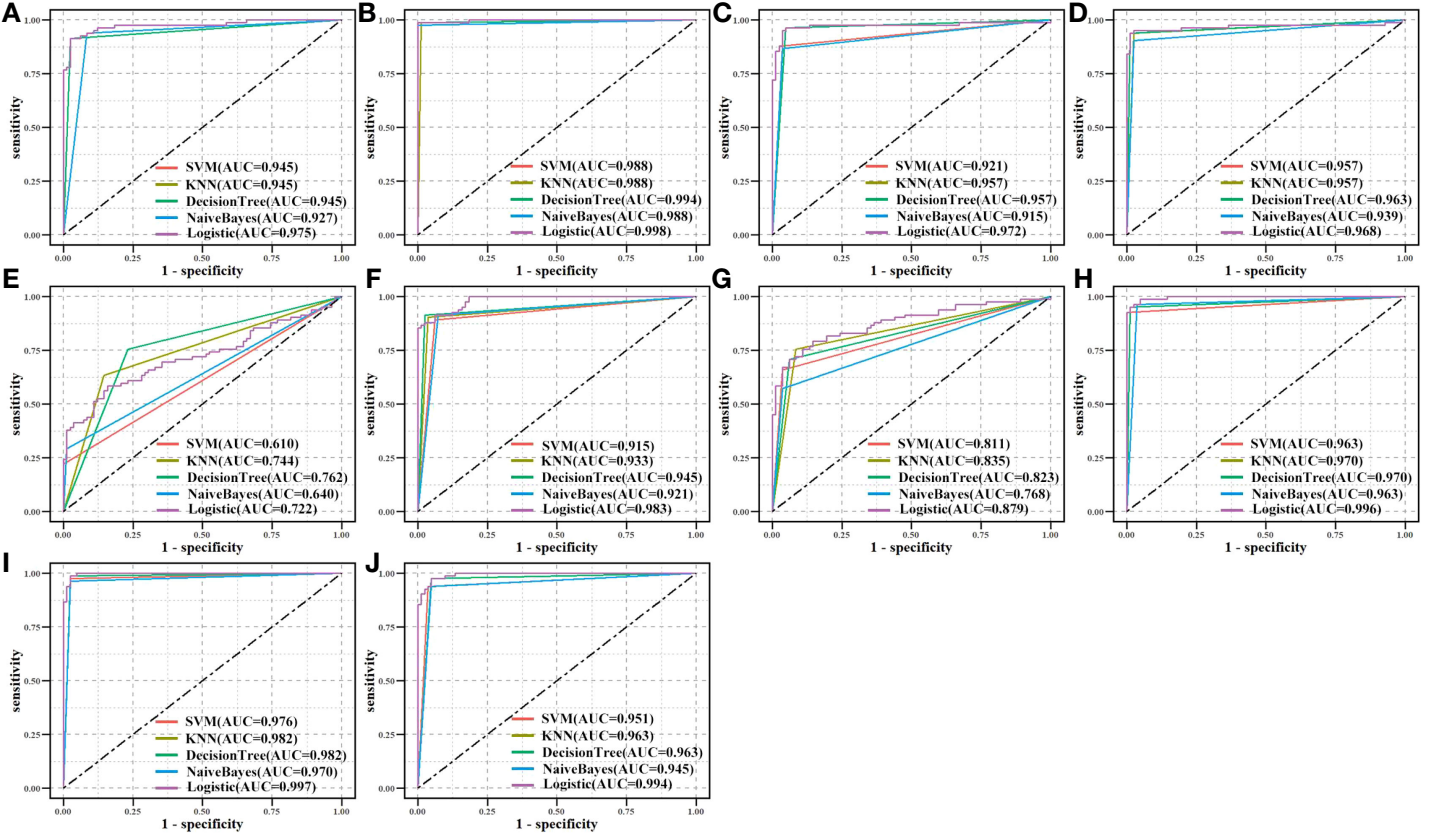
Validation of biomarkers’ classification effectiveness assessment using different classifier models in the CPTAC independent dataset. **(A)** CAT, **(B)** CAV1, **(C)** ENO1, **(D)** GAPDH, **(E)** GPX2, **(F)** GPX3, **(G)** NQO1, **(H)** P4HB, **(I)** PDIA4, and **(J)** PDIA6.

An independent CPTAC dataset was used to validate biomarkers’ classification effectiveness for tumor and normal samples (**Figure 9**). Eight biomarkers (AUC > 0.900), NQO1 (AUC > 0.750), and GPX2 (AUC > 0.600) in all machine learning methods showed good classification performance, both on TCGA and CPTAC datasets. The results demonstrated the potential diagnostic values of all biomarkers for LUAD.

Finally, a literature review was carried out by searching the PubMed database for all publications published in English for the relevant biomarkers for LUAD. All 10 biomarkers had been validated in the literature as potential prognostic markers for LUAD (61–69). Four metabolites were directly connected to biomarkers in the subnetwork (**Figure 5B**). Cancer development may be linked to alterations in GPX2 and GPX3 activities, which were associated with glutathione (C00051), oxidized glutathione (C00127), and hydrogen peroxide (C00027). Glutathione is a specific tripeptide and engages in numerous intercellular activities. Cancer cells with high glutathione levels are resistant to chemotherapy (70). Oxidized glutathione (GSSG) is formed by glutathione peroxidases (GPXs). The GSSG content rises due to GPX3 overexpression, in turn, increasing glutathione levels (71). Hydrogen peroxide accelerates cell proliferation and decreases rapamycin-induced autophagy along with increasing intracellular reactive oxygen species (ROS) levels. Elevated intracellular levels of hydrogen peroxide and ROS lead to PTEN inactivation and AKT/mTOR pathway activation, which prevents autophagy and promotes LUAD cell growth (72). NQO1 is intimately connected to NADPH (C00005) and reduces the malignant characteristics of LUAD (73). miR-485-5p targets NADPH to oxidize NQO1 and inhibit PI3K/Akt, thus counteracting the inhibitory effect of NQO1 on the malignant phenotype of LUAD cells, thereby preventing LUAD cell proliferation and migration.

## 4 Discussion

LUAD is the most widely occurring subtype of lung cancer and among the major causes of death due to cancers. Cancer is a metabolic disease, and metabolic reprogramming is a result of certain oncogenic changes that promote cancer development and progression through complex interactions with the tumor ecosystem (74). Given this background, we constructed an MMI network to understand cancer metabolism comprehensively. As a result, 10 metabolism-related biomarkers were identified from a metabolic perspective using the DNN model in the MMI network. The survival prognosis and classification effectiveness of biomarkers were confirmed by the literature and data from TCGA, CPTAC, and GEO. ENO1, GAPDH, NQO1, PDIA4, and PDIA6 may serve as potential targets for cancer therapy (69, 75–77).

To strengthen our findings of the 10 metabolism-related biomarkers, we conducted differential expression analysis and survival analysis in the datasets derived from eight different cancer cohorts (including LUSC, BRCA, CESC, KICH, LIHC, PAAD, PRAD, and STAD) from TCGA (**Table S6**). The results of the differential expression analysis revealed that the expression patterns of the 10 biomarkers differed among seven cancers (including BRCA, CESC, KICH, LIHC, PAAD, PRAD, and STAD) compared to LUAD. Additionally, survival analysis indicated that the prognostic significance of the 10 biomarkers was statistically insignificant (p > 0.05) for the majority of these seven cancers. These observations suggested that the identified biomarkers in LUAD were not biomarkers for these seven cancers and were not consistently regulated in these seven cancers. LUAD and lung squamous cell cancer are the two predominant subtypes of NSCLC, and so, a comparison of the 10 biomarkers was performed in these two subtypes (**Tables S6**, **S7**). CAT, ENO1, NQO1, P4HB, and PDIA6 were unique to LUAD, while CAV1, GAPDH, GPX2, GPX3, and PDIA4 exhibited consistent trends in differential expression in both LUAD and lung squamous cell cancer, significant prognostic survival prediction (p<0.05), and excellent classification effectiveness. These mRNAs may serve as potential biomarkers for NSCLC. Furthermore, we conducted a differential analysis for biomarker expression in different stages of LUAD samples from TCGA (**Table S8**). GAPDH and P4HB were significantly different (p<0.05) between stages I and II, while ENO1, GAPDH, and PDIA6 were significantly different (p<0.05) between stages I and III and CAT, ENO1, GAPDH, P4HB, and PDIA6 were significantly different (p<0.05) between stages I and II+III. These are potential biomarkers for staging patients with LUAD.

Using four public databases (KEGG, Reactome, Human-GEM, BRENDA), we constructed an MMI network and it was found to be comprehensive and reliable. In the network, we established a metabolism-related mRNA DNN model, and candidate mRNAs were identified more precisely using the DNN model along with weight values. This was due to the inherent advantage of the DNN model to change the multidimensional weights of each feature during learning and describe intricate relationships between mRNAs. Therefore, it was more accurate at filtering features than conventional machine learning techniques. Moreover, when using the DNN model, the learning state of the model is usually assessed based on the decrease in the validation loss rate and the training loss rate during the learning process. In this situation, two phenomena are commonly encountered during deep learning: overfitting and underfitting. When the model was overfitting (**Figure S1A**), model regularization and reducing the learning rate are common optimization techniques; whereas, when the model was underfitting (**Figure S1B**), it is necessary to reduce both the learning rate and the batch size to improve the generalization ability. If both the validation loss rate and training loss rate converge to 0 (**Figure S1C**), no further training is required and the model is more suitable for generalization. Based on these considerations and the sample size of the TCGA dataset used in this study, batch size = 16, epoch = 2000, and learning rate = 0.00001 were chosen.

**Figure 10:**
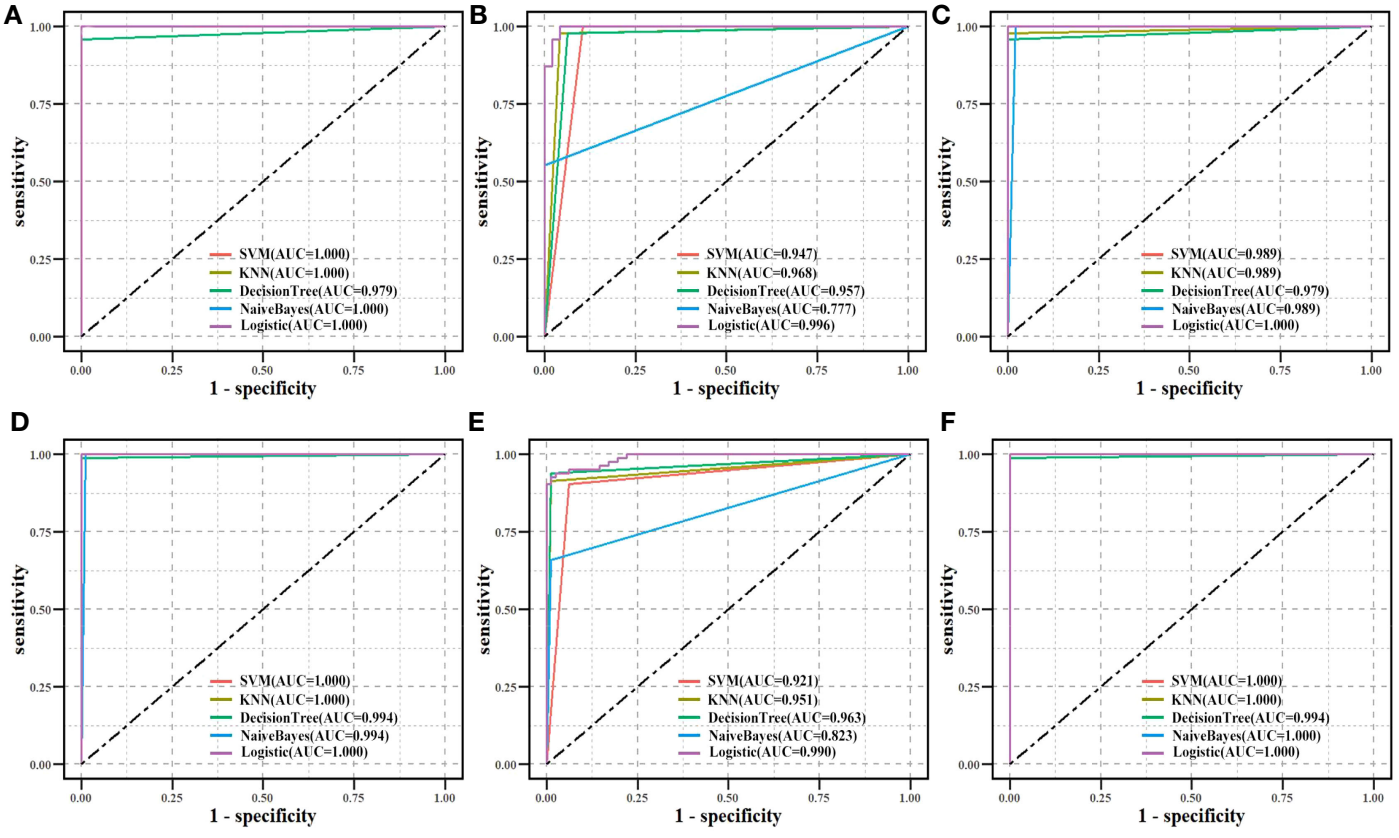
Biomarkers’ classification effectiveness assessment using different classifier models in TCGA. **(A)** Ten biomarkers were assessed as an overall factor. **(B)** GPX2, GPX3 as a factor. **(C)** CAV1, ENO1, NQO1, and P4HB as a factor. Biomarkers’ classification effectiveness assessment using different classifier models in CPTAC. **(D)** Ten biomarkers were assessed as an overall factor. **(E)** GPX2, GPX3 as a factor. **(F)** CAV1, ENO1, NQO1, and P4HB as a factor.

The identified biomarkers in this study were enriched in metabolic function classes and pathways in LUAD, and can potentially characterize a patient’s dysfunction. Hence, the classification effectiveness of ten biomarkers which was assessed overall was based on GPX2 and GPX3 as factors from the enriched pathways and CAV1, ENO1, NQO1, and P4HB as factors from functional classes to determine whether a patient had cancer. The 594 samples (including 535 tumor samples and 59 normal samples) from TCGA were split into a training set and a testing set in a ratio of 8:2. The independent CPTAC dataset was used for validation in the same way (**Figure 10**). Both in the TCGA dataset and the CPTAC independent dataset, the majority of machine learning approaches showed good classification effectiveness (AUC > 0.800), highlighting the potential diagnostic values of biomarker combinations for LUAD samples.

To examine the synergistic effect of the 10 markers on the prediction of patient prognosis, Lasso-penalized Cox regression (78) was conducted to screen biomarkers for building a risk model. The optimal value of the Lasso penalty parameter, λ, was determined as 0.0078 through 10‐fold cross-validation (**Figure S2**). Then, to select the best model as the risk model (79), the outcomes of the Lasso analysis were evaluated using multifactorial Cox regression analysis. CAV1, ENO1, and GAPDH (which were defined as risk mRNAs) were used with a p-value threshold of 0.05 (**Table 4**), and the final risk model was constructed as follows:

**Table 4:** Multivariate Cox regression analyses.

	HR	95% CI	p-value
ENO1	1.0008	1.0001-1.0015	0.031
CAV1	1.0023	1.0003-1.0043	0.022
GAPDH	1.0006	1.0003-1.0008	<0.001


$$RiskScore=\left(0.0024\times EXP_{CAV1}\right)+\left(0.0009\times EXP_{ENO1}\right)+\left(0.0005\times EXP_{GAPDH}\right).$$

For all tumor samples in TCGA, risk scores were computed and divided into high‐ and low‐risk groups using the median risk score as the cutoff. Distributions of risk scores, survival statuses, and survival curves (**Figure S3A**) are shown. To validate the risk model, GSE36471, GSE42127, GSE68465, and GSE72094 were used as the validation datasets. Risk scores were computed and high‐ and low‐risk groups were obtained (**Figures S3B–E**). Patients in the risk-score-high group died more and had slightly shorter survival than those in the risk-score-low group. Kaplan-Meier curves illustrated patients with LUAD in the risk-score-high group had a worse overall survival rate than those in the risk-score-low group in all five datasets. DEmRNAs between high- and low-risk groups were identified and highly expressed DEmRNAs in the high-risk group were enriched in the cell cycle, including the mitochondrial cell cycle process, cell division, and regulation of the cell cycle process. Mounting evidence shows that cancer metabolism is intertwined with cell cycle regulatory mechanisms. Therapy aimed at cell cycle machinery thereby inhibits cancer cell division while also reversing malignant cell metabolism (80). Hence, the outcomes of the enrichment analysis supported the risk model which was based on metabolism-related biomarkers and confirmed the above-mentioned mRNAs’ distinct roles in metabolism. The classification effectiveness of the risk model for high‐ and low‐risk score groups in the samples from TCGA (**Figure S4A**) and GSE36471, GSE42127, GSE68465, and GSE72094 (**Figures S4B–E**) was good (AUC > 0.750). Consequently, the risk model had a good prognostic predictive value and classification effectiveness for LUAD, which also proved the reliability of these biomarkers.

## 5 Conclusions

In conclusion, from the metabolism perspective, we constructed the MMI network and the DNN model and successfully applied them to predictions for LUAD. The importance of the 10 identified metabolism-related biomarkers was confirmed for prediction of survival and classification effectiveness. This integrated method and approach may offer a novel perspective to identify biomarkers for other malignancies.

## Data availability statement

The original contributions presented in the study are included in the article/**Supplementary Material**, further inquiries can be directed to the corresponding authors.

## Author contributions

LF: Formal Analysis, Investigation, Methodology, Software, Visualization, Writing – original draft, Writing – review & editing. JL: Data curation, Writing – review & editing. CY: Data curation, Writing – review & editing. ML: Validation, Writing – review & editing. ZZ: Writing – review & editing. SQ: Writing – review & editing. WL: Funding acquisition, Writing – review & editing. XW: Conceptualization, Data curation, Writing – review & editing. LC: Conceptualization, Funding acquisition, Supervision, Writing – review & editing.
