# Supplementary figures and images for: Deep neural network for discovering metabolism-related biomarkers for lung adenocarcinoma
Source: Front Endocrinol (Lausanne). 2023 Oct 25;14:1270772. doi: 10.3389/fendo.2023.1270772 (PMC10634586; doi:10.3389/fendo.2023.1270772)

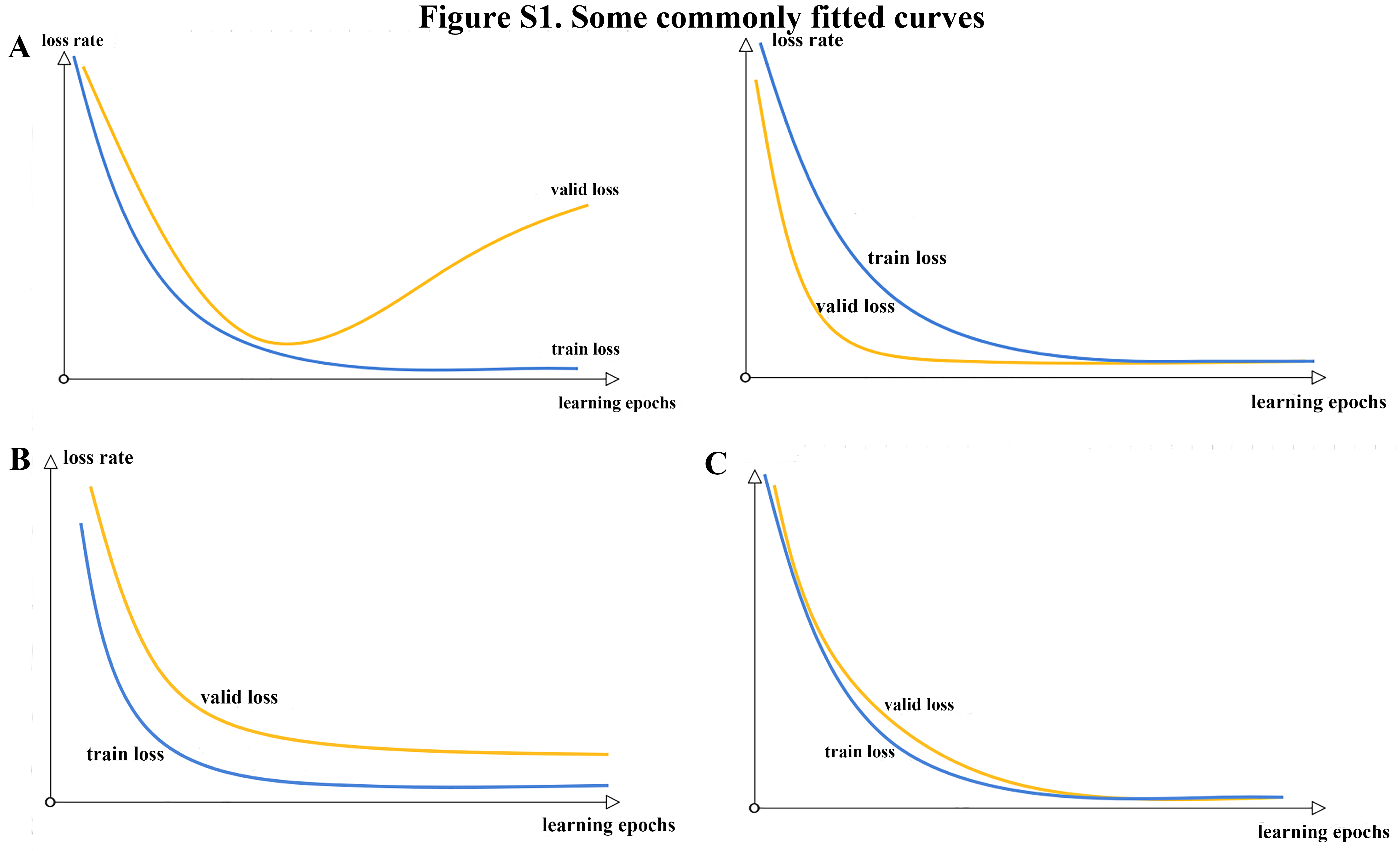

Supplement: Supplementary file 2 [file DataSheet_2.zip › Figure S1. Some commonly fitted curves.jpg]

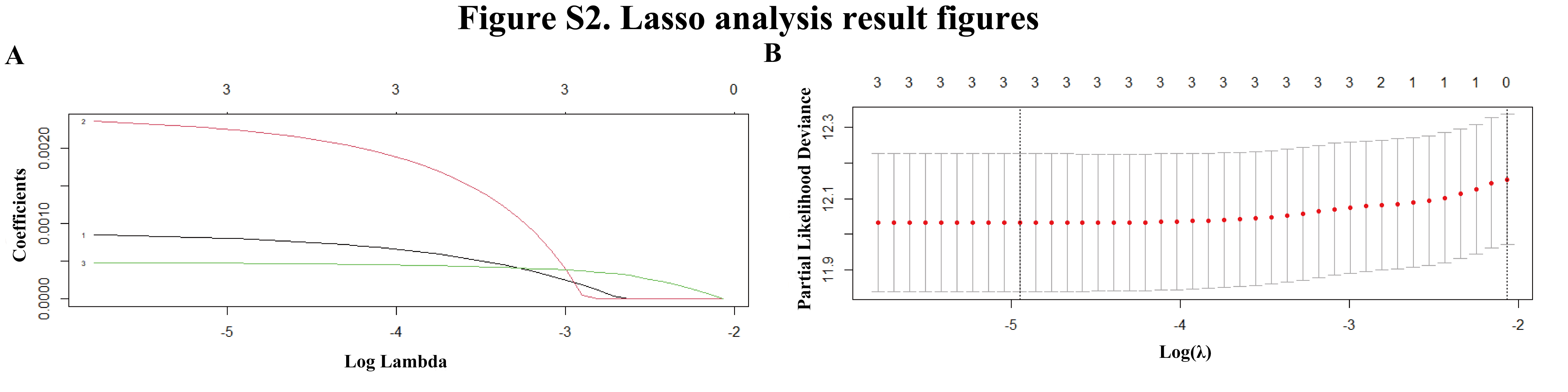

Supplement: Supplementary file 2 [file DataSheet_2.zip › Figure S2. Lasso analysis result figures.jpg]

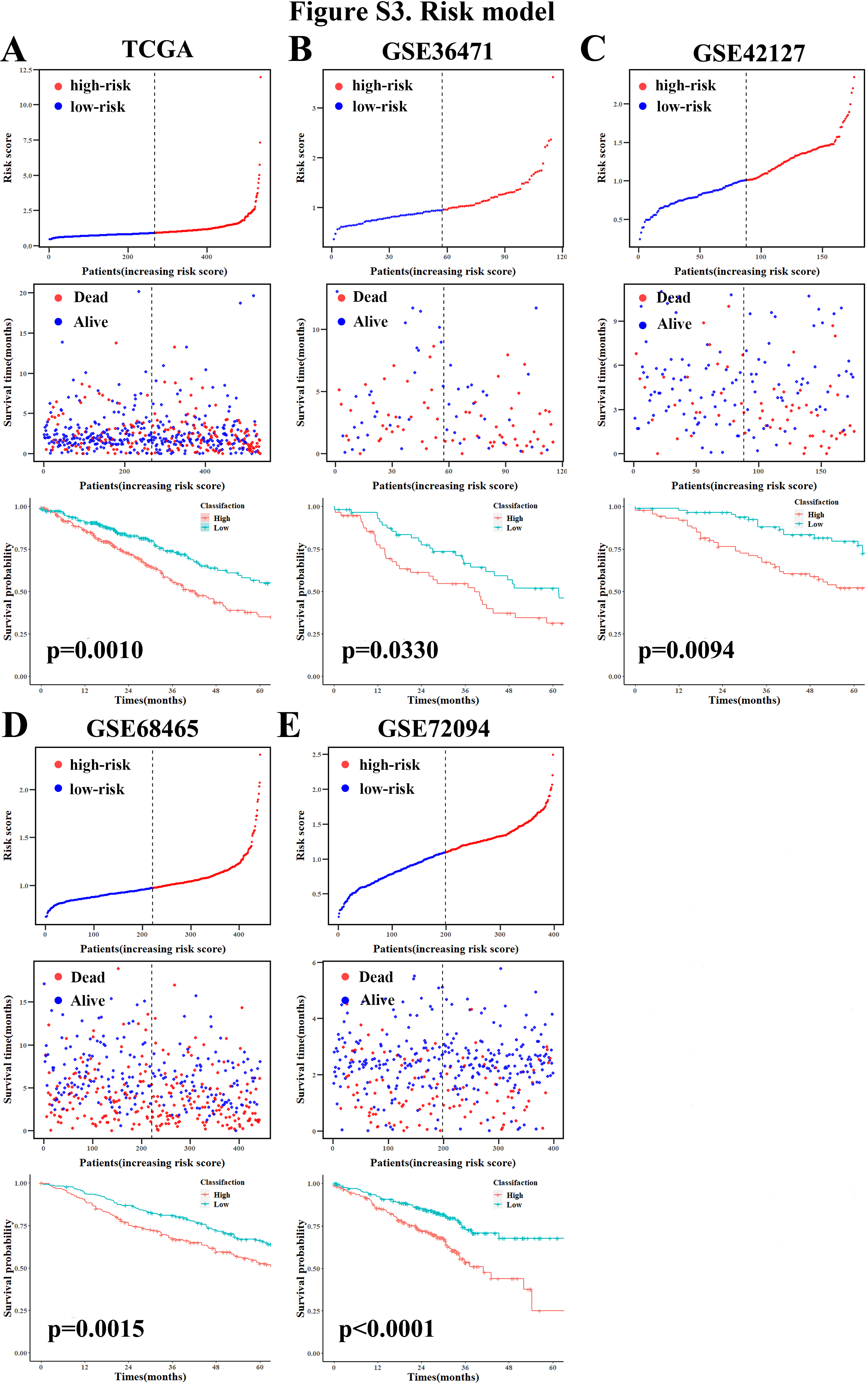

Supplement: Supplementary file 2 [file DataSheet_2.zip › Figure S3. Risk model.jpg]

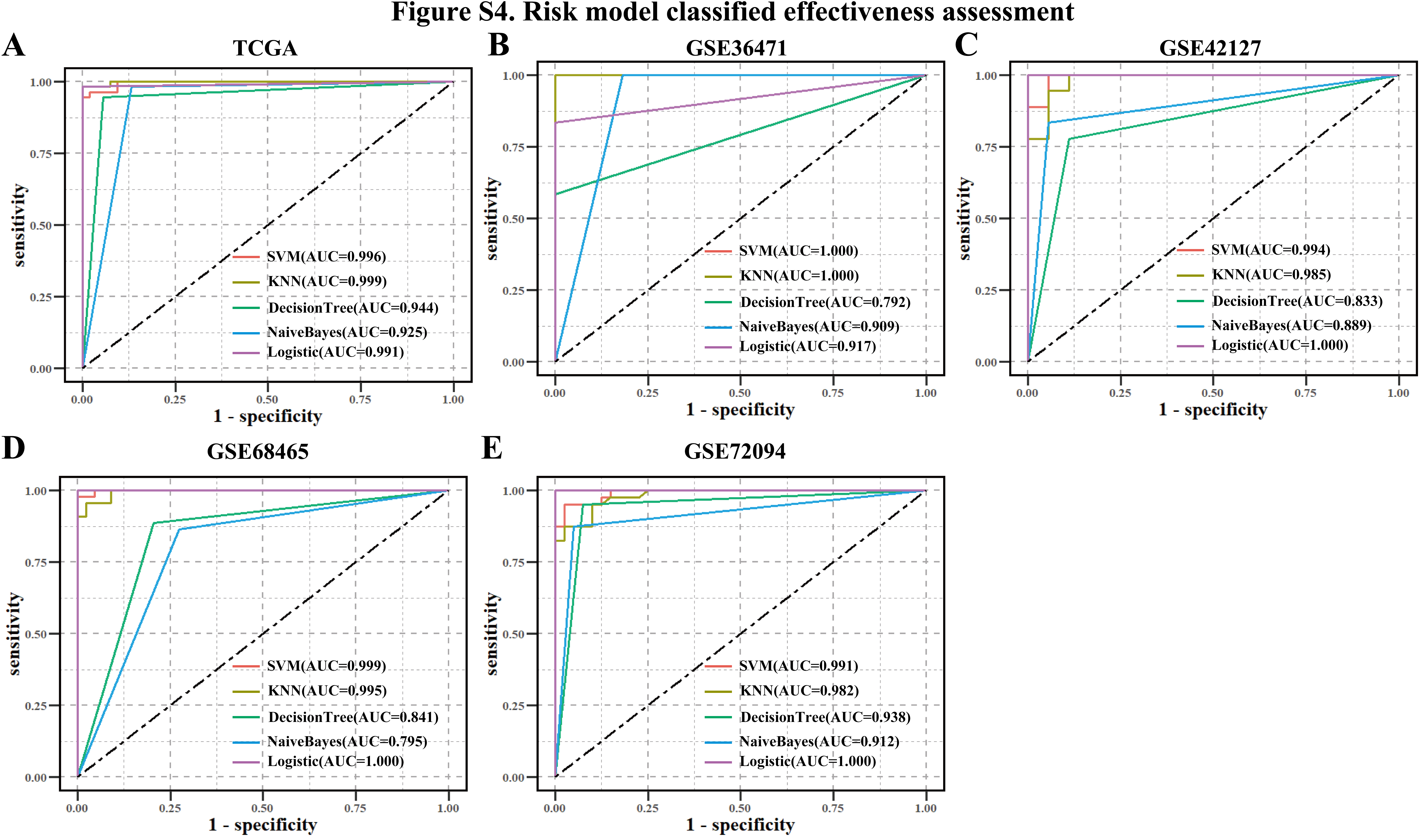

Supplement: Supplementary file 2 [file DataSheet_2.zip › Figure S4. Risk model classified effectiveness assessment.jpg]
